# Advancing the sexual and reproductive health and human rights of women living with HIV: a review of UN, regional and national human rights norms and standards

**DOI:** 10.7448/IAS.18.6.20280

**Published:** 2015-12-01

**Authors:** Rajat Khosla, Nuna Van Belle, Marleen Temmerman

**Affiliations:** Department of Reproductive Health and Research, World Health Organization, Geneva, Switzerland

**Keywords:** sexual and reproductive health, HIV, women living with HIV, human rights

## Abstract

**Introduction:**

The right to sexual and reproductive health (SRH) is an essential part of the right to health and is dependent upon substantive equality, including freedom from multiple and intersecting forms of discrimination that result in exclusion in both law and practice. Nonetheless, general and specific SRH needs of women living with HIV are often not adequately addressed. For example, services that women living with HIV need may not be available or may have multiple barriers, in particular stigma and discrimination. This study was conducted to review United Nations Human Rights Council, Treaty Monitoring Bodies and Special Rapporteur reports and regional and national mechanisms regarding SRH issues of women living with HIV. The objective is to assess areas of progress, as well as gaps, in relation to health and human rights considerations in the work of these normative bodies on health and human rights.

**Methods:**

The review was done using keywords of international, regional and national jurisprudence on findings covering the 2000 to 2014 period for documents in English; searches for the Inter-American Commission on Human Rights and national judgments were also conducted in Spanish. Jurisprudence of UN Treaty Monitoring Bodies, regional mechanisms and national bodies was considered in this regard.

**Results and discussion:**

In total, 236 findings were identified using the search strategy, and of these 129 were selected for review based on the inclusion criteria. The results highlight that while jurisprudence from international, regional and national bodies reflects consideration of some health and human rights issues related to women living with HIV and SRH, the approach of these bodies has been largely *ad hoc* and lacks a systematic integration of human rights concerns of women living with HIV in relation to SRH. Most findings relate to non-discrimination, accessibility, informed decision-making and accountability. There are critical gaps on normative standards regarding the human rights of women living with HIV in relation to SRH.

**Conclusions:**

A systematic approach to health and human rights considerations related to women living with HIV and SRH by international, regional and national bodies is needed to advance the agenda and ensure that policies and programmes related to SRH systematically take into account the health and human rights of women living with HIV.

## Introduction

Protection of the sexual and reproductive health (SRH) and human rights of women living with HIV/AIDS is fundamental to their dignity, health and wellbeing [1]. However, HIV continues to be a leading cause of death among women of reproductive age worldwide. To address this situation, the global HIV response must fully recognize the significant role that gender inequality and violation of human rights plays in increasing women and girls’ vulnerability to HIV [2].

Everyone has the equal rights concerning their SRH. However, women living with HIV/AIDS require special protection in this regard. HIV infection accelerates the natural history of some reproductive illnesses and increases the severity of others [1]. Moreover, infection with HIV has serious effects on the sexual health and wellbeing of women [1]. Studies demonstrate that women and girls living with HIV have less access to prevention, treatment, care and support [3]. There is a growing realization that protection and promotion of SRH and rights, including through improved and sustained investment in women and girls living with HIV, can help countries move towards universal access to HIV prevention, treatment, care and support services [4].

For decades, organizations and groups of women living with HIV, such as the Salamander Trust, the Athena Network, the Global Network for and by People Living with HIV and the International Community of Women Living with HIV/AIDS, have been at the forefront of development of research and normative standards in relation to the SRH and human rights of women living with HIV. The work of these organizations has not only helped in the galvanization of support for the development of normative standards in this regard, but also in the improvement of prevention of treatment and care for women living with HIV [5].

The right to SRH is an essential part of the right to health and is dependent upon substantive equality, including freedom from multiple and intersecting forms of discrimination that exacerbate exclusion in both law and practice [6]. Multiple reports highlight the fact that general and specific SRH needs of women living with HIV are often not adequately addressed [7–9]. For example, the SRH services that women living with HIV need may not be available or these women may face multiple barriers, in particular stigma and discrimination, in accessing existing services (see Supplementary Table 1) [8,10–13].

This study was conducted to review findings of international, regional and national bodies regarding SRH issues of women living with HIV. This study was conducted with the objective to assess key areas of progress and possible gaps in relation to normative development of human right standards by United Nations, regional and national human rights bodies regarding the SRH of women living with HIV.

## Method

The starting point for this study is the UN Population Fund (UNFPA) and the World Health Organization (WHO) guidelines, *Sexual and reproductive health of women living with HIV/AIDS* (2006) [1]. The recommendations on care, treatment and support for women living with HIV/AIDS and their children were used to define the search strategy for this study.

The study reviewed relevant findings of the UN Human Rights Council, Treaty Monitoring Bodies and Special Rapporteurs (these included reports, concluding observations and general comments) in relation to normative developments regarding the human rights of women living with HIV in the context of SRH. The review was done for findings covering 2000 to 2014 for documents in English; searches were also conducted in Spanish for the Inter-American Commission on Human Rights (IACHR/CIDH) including the site of the Organization of American States (OAS) and national judgments. The period of 2000 to 2014 was selected with the view that this is period in which the UN Committee on Economic, Social and Cultural Rights General Comment No. 14 on the right to health laid down the framework on health and human rights [14].

The review process was divided into three stages.

First, an international normative review was undertaken. This step included reviews of findings of the Human Rights Council, Treaty Monitoring Bodies and Special Rapporteur reports. Four databases were therefore used: the OHCHR Universal Human Rights Index; bayefsky.com; the University of Minnesota Human Rights Library; and the Universal Periodic Review (UPR). The list of search terms and databases used for the purposes of this review are included in Supplementary Annex 1. Findings include results from documents of the Committee against Torture; Committee on the Elimination of Discrimination against Women; Committee on the Rights of the Child; Committee on Economic, Social and Cultural Rights; Committee on Civil and Political Rights; Special Rapporteur on Health; Special Rapporteur on Mental and Physical Health; Special Rapporteur on Violence against Women; and UPR Working Group.

Second, a regional normative review was undertaken. This included reviews of findings from resolutions and decisions of regional human rights bodies. Sites from the IACHR/CIDH, including the site of the OAS; the African Commission of Human and Peoples’ rights (including the site of the African Union); and the European Commission of Human Rights (including the site of the Council of Europe) were reviewed. The list of search terms and databases used for this review are included in Supplementary Annex 2.

Third, a national normative review was undertaken. This step included reviews of data extracted from national judgments. Different databases were consulted, including LexisNexis, the Treatment Action Campaign database, the South African Legal Information Institute database, the Center of Reproductive Health database, the Global Health and Rights database and national databases with official publications of judgments. References to judgments were also found in the United Nations Development Programme (UNDP) *Compendium of Judgments for Judicial Dialogue on HIV, Human Rights and the Law in East and Southern Africa* of October 2013, the UNDP *Compendium of Judgments for Judicial Dialogue on HIV, Human Rights and the Law in Asia and the Pacific* of June 2013 and the UNAIDS *Judging the Epidemic: A Judicial Handbook on HIV, Human Rights and the Law* of May 2013. Subsequently, references were used to locate the original decisions, and data were directly extracted from official publications. Where the judgments could not be found, the data extraction table (Supplementary Table 1) indicates this.

In terms of the inclusion criteria, a decision was made to include not only findings where human rights bodies had *explicitly* made observations on the SRH of women living with HIV, but also those that were *implicitly* dealing with these issues even if not specifically addressing the nine agreed-upon human rights dimensions found in the WHO's *Ensuring Human Rights in the Provision of Contraceptive Information and Services* (2014):

1. Equality and non-discrimination (alternate terms: *reduce discrimination, reduce criminalization, combat negative social and cultural attitudes, stigma, prejudice, [domestic] violence, gender inequality*)
2. Participation (alternate terms: *involvement, advocacy, influence*)
3. Privacy and confidentiality
4. Informed decision-making (alternate terms: [*direct*] *consent, choice, coerced, forced, informed, comprehensible*)
5. Availability (alternate terms: *make available, provide, exist*)
6. Accessibility (alternate terms: *access, receive, affordable, eligible*)
7. Acceptability (alternate terms: *conscientious objection, medical ethics, human rights sensitivity*)
8. Quality of services (alternate terms: *proper medical care, adequacy*)
9. Accountability (alternate terms: *liability, responsibility, calling upon state parties, enforcement, legal measures*) [15].

Findings that dealt with issues related to HIV and SRH without a specific focus on issues related to women living with HIV were excluded. Similarly, findings that looked at SRH issues of women without a specific focus on women living with HIV were also excluded. Further, in order to capture the widest array of relevant observations to be found in the normative work, search terms also included *stigma*, *respect* and *disrespect*, as well as *choice*.

Data on determinants of health were included to a limited extent.

## Results and discussion

**Figure 1:**
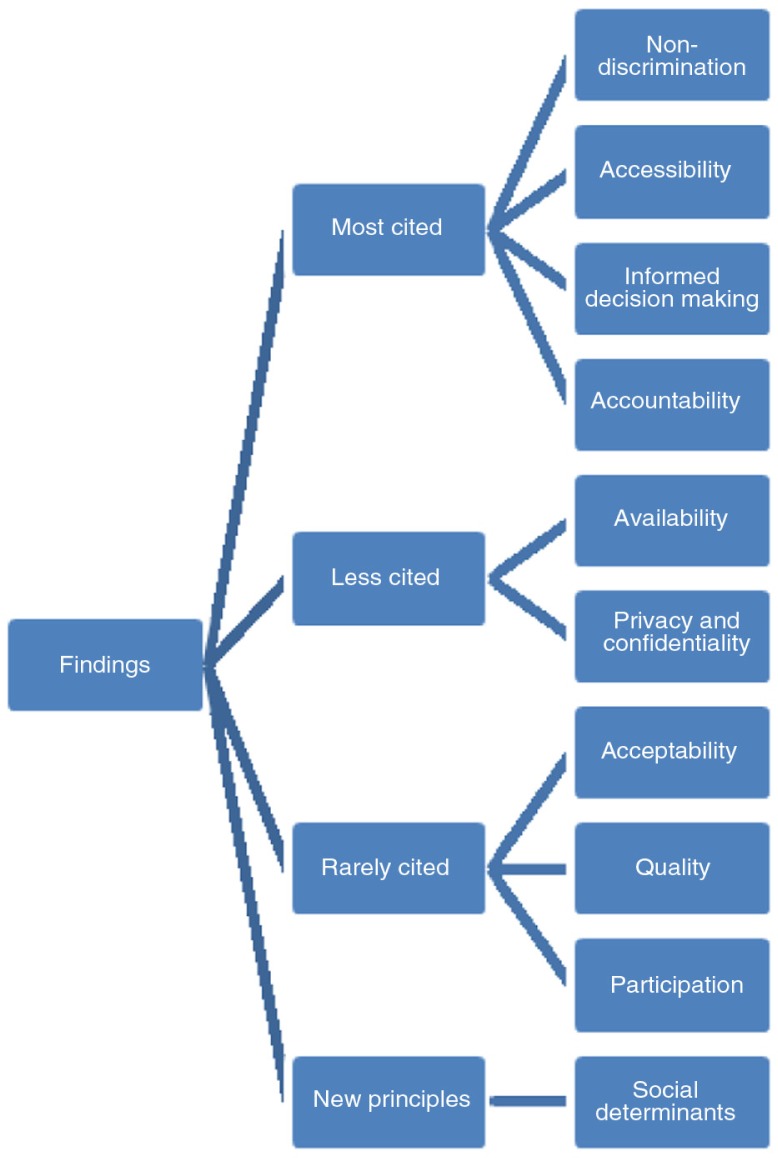
Review findings.

The principles that are most discussed by international, regional and national bodies or courts, in the context of SRH of women living with HIV, are non-discrimination (see Box 1), accessibility, informed decision-making and accountability (see Supplementary Table 1 for survey findings; see also Figure 1).

**Box 1: Key definitions**

1. The *principle of non-discrimination* obliges states to guarantee that human rights are exercised without discrimination of any kind based on, *inter alia*, colour, sex, language, religion, political or other opinion, national or social origin, property, birth or other status, such as disability, age, marital and family status, sexual orientation and gender identity, health status, place of residence and economic and social situation.
2. The *principle of accessibility* implies that health facilities, goods and services have to be accessible to everyone without discrimination.
3. The *principle of informed decision-making* implies giving each person the choice and opportunity to make autonomous reproductive choices. The principle of autonomy, expressed through free, full and informed decision-making, is a central theme in medical ethics and is embodied in human rights law.
4. The *principle of accountability* implies that generally states’ legal, policy and programmatic frameworks and practices should be in line with international, regional and national human rights standards. The establishment of effective accountability mechanisms is intrinsic to ensuring that the choices of individuals are respected, protected and fulfilled. Effective accountability requires individuals to be aware of their entitlements with regard to SRH and of the mechanisms available to them.

A total of 236 findings were identified based on the search strategy. Based on the inclusion criteria, 129 findings were selected, the full text was reviewed and data were extracted. The results of the review were classified according to the nine human rights principles and arranged on the basis of them being *most cited*, *less cited* or *rarely cited*. The authors manually reviewed the findings to ascertain how these principles had been dealt with and the frequency with which these principles were referred to in the human rights normative developments related to women living with HIV and SRH. For the purposes of this classification, principles cited >10 times were classified as *most cited*, principles cited <10 times as *less cited* and principles cited <5 times as *rarely cited*. In addition, principles that did not feature under the nine developed by the WHO but were frequently cited by the UN and/or regional and national human rights bodies were also noted.

### Most-cited human rights principles in relation to women living with HIV and SRH

#### The principle of non-discrimination

The review of international, regional and national jurisprudence of normative standards found that the most-cited human rights principle in relation to the SRH of women living with HIV is the need to combat discrimination and violence against women living with HIV. The findings from various human rights bodies refer to the need to eliminate discrimination against women, girls and adolescents living with HIV through challenging gender inequality, stereotypes, stigma, prejudice and violence. According to the findings, discrimination toward women living with HIV occurs primarily within families, communities and healthcare facilities.

Furthermore, violence is highlighted in the findings of human rights bodies as a central concern with regard to the SRH of women living with HIV. The findings highlight the need to eliminate violence by addressing gaps in legislation and policy. Violence or fear of violence is identified as a prime barrier to HIV testing and disclosure of a women's seropositive status. Different types of violence (psychological and physical) are mentioned, including sexual violence, prejudicial traditional or customary practices, coercion or abuse, early and forced marriage, fear of conflict with partners, forced vaginal examinations, mandatory testing and involuntary sterilization. The findings emphasize that women living with HIV are more likely to experience violence than men living with HIV [16]. In this context, it is important to highlight the findings on the need to empower women, support their economic independence and protect their fundamental rights and freedoms, including their SRH rights.

Human rights bodies also cite stigma and prejudice as leading obstacles to the enjoyment of SRH by women living with HIV. They impede the access of women living with HIV to justice and severely limit or deny the enjoyment of these women's SRH.

The findings of human rights bodies further identify gender inequalities and stereotypes as a major issue. The vulnerability of women and girls living with HIV/AIDS is a major human rights challenge because of the effect of inequality between the sexes. Mothers are held solely responsible for infecting their children. Women are held responsible for HIV transmission by the very person who infected them, and HIV-positive men sometimes believe that they have the right to maintain the pleasure of unprotected sex [17]. The findings also highlight the relationship between violence and gender stereotypes. The findings emphasize the need to combat discrimination and violence by addressing gaps in legislation and policy, putting programmes into place and implementing initiatives [18].

#### The principle of accessibility

Accessibility of information and services related to SRH remains a challenge for women living with HIV. The findings of human rights bodies indicate issues of discrimination in accessibility by women living with HIV to SRH information and services, in particular in family planning, pregnancy and childcare. Most findings are related to treatment of women in their reproductive years and some to female children; however, some categories of women, such as women without children and older women, are hardly taken into account. Nevertheless, one particular reference stresses the need for equitable access to SRH care throughout the lives of women living with HIV [19] and is one of the few examples whereby human rights bodies have made explicit reference to the importance of access to treatment throughout women's lives.

Findings of human rights bodies also point to the physical inaccessibility of most rural and marginalized women living with HIV to healthcare services, which leads to delays and difficulties in the utilization of adequate information and services. Furthermore, findings highlight that migrant women living with HIV also face social, language, legal and financial barriers and are exposed to the risk of inaccessibility to services when submitted to deportation [20,21].

Economically accessible information and SRH services, such as HIV testing, counselling, contraceptives and antiretroviral (ARV) treatment, are often supported, according to the findings of human rights bodies. However, as with the physical accessibility of services, all these references primarily focus on pregnant women's economic accessibility to services.

#### The principle of informed decision-making

Women living with HIV are often sterilized without their knowledge or consent, and there is a need for education about the effects of sterilization and the alternatives available [22]. In addition, pregnant women living with HIV are often advised or pressured to terminate their pregnancies [8]. The review of findings of human rights bodies highlights the need for these women to be informed about ARV medication during pregnancy and delivery and after birth. Findings also highlight that many women are submitted to mandatory HIV testing and therefore emphasize the need for free and informed consent with regard to all medical procedures [23]. Within this context, a large number of findings relate to the lack of information on prevention of mother-to-child transmission (PMTCT) of HIV.

#### The principle of accountability

Findings of human rights bodies refer to the need to encourage a policy, legal and social environment that promotes human rights for women living with HIV, ensuring the full recognition of their SRH and rights. Findings point towards the need to address existing gaps in HIV-related legislation and policy and further highlight the need to effectively use parliamentary processes. National mechanisms such as commissions, courts, legislation and coordinated strategies must be strengthened to protect, enforce and monitor the human rights of women living with HIV. Implementation and enforcement of protection in law for women living with HIV remains a challenge. The issue of criminalization of HIV transmission to others and, in the case of pregnant women, to the foetus is also emphasized in several human rights bodies’ findings [24,25].

Furthermore, the findings of human rights bodies points towards evidence that women living with HIV face multiple forms of discrimination with regard to access to justice. Findings highlight the need to put reinsertion programmes into place for women living with HIV who are victims of discrimination.

### Less-cited human rights principles in relation to women living with HIV and SRH

The results from the review of findings from human rights bodies reflect a primary focus on issues related to non-discrimination, accessibility, informed decision-making and accountability in international, regional and national jurisprudence related to women living with HIV and SRH. Some additional references are also found for other key health and human rights considerations, in particular availability and privacy and confidentiality.

#### Principle of availability

Within the principle of availability, the human rights bodies’ findings primarily focus on PMTCT [26–28]. A lot of references are made to the availability of sufficient quantity of goods and services and programmes [29]. The availability of goods focuses primarily on ARV treatment for PMTCT. The findings also refer to the availability of sufficient and regular paediatric ARV treatment and the availability of ARVs in prisons and public hospitals. The availability of services is also primarily dealt with in the context of PMTCT. Within this context, the findings underline the need for prevention of unintended pregnancies and for appropriate antenatal, delivery and postpartum care, including counselling on infant feeding options.

Furthermore, the findings underline the importance of the integration of HIV/AIDS services in SRH care and *vice versa*. Most findings relate to the importance of integrating HIV/AIDS issues in SRH programmes [25,30,31].

#### Privacy and confidentiality

Findings of human rights bodies on confidentiality and privacy primarily deal with the disclosure of women's HIV status. There is a lack of confidentiality in health facilities, schools, prisons and courts. Test results are made available to husbands, friends, families and the community at large.

### Least-cited human rights principles in relation to women living with HIV and SRH

The principles of acceptability, quality and participation are least dealt with in international, regional and national human rights jurisprudence related to women living with HIV and SRH. Whereas hardly any references are found regarding principles related to acceptability and quality, there are some references related to participation. Participation is primarily emphasized with respect to women living with HIV, as well as civil society at large, which must be encouraged to participate in the development and implementation of national policies and actions. Religious communities are encouraged to include provisions on premarital HIV counselling and testing in their by-laws.

### Newly cited principles: determinants of health

The analysis of jurisprudence also points to some references to the experience of discrimination faced by women living with HIV in access to housing, education, employment, healthcare and justice [32]. These principles are in addition to the nine health and human rights principles of WHO and are noted here for their relevance to the issue of women living with HIV and SRH.

### Gaps and challenges

These findings clearly illustrate that while international, regional and national bodies have been considering issues related to health and human rights of women living with HIV and SRH, various health and human rights considerations are often not systematically addressed.

The study identified some key limitations in the way that UN human rights mechanisms have dealt with issues related to women living with HIV and SRH. These include the following issues.

#### Ambiguity around the subject of women living with HIV

An overwhelming number of references to mother-to-child transmission or vertical transmission of the virus were found in the review, and although the prevalence of HIV among women is said to be “particularly concerning” in its own right, the focus was on *its potential to transmit the disease through child rearing*. In addition, many items promoting SRH for women living with HIV rely on vague terms. For example, some documents provide that states should “eliminate discrimination against women and persons living with HIV.” This statement leaves its subject unclear. Should member states not discriminate against women *and* persons with HIV (separate categories)? Or rather, should the statement be understood as non-discrimination against “women *and/or* persons living with HIV?” This ambiguity in the way the issues have been dealt with obscures meaning and impact of the findings. One cannot assume that lists of disadvantaged social categories incorporate persons at the intersection.

#### Ambiguity around the subject of sexual and reproductive health

Often, references to the “prevention and future control of HIV” and “human rights guarantees” for women living with HIV are mixed with specific human rights related to women living with HIV and SRH. Despite the fact that SRH *is* a human right, not all persons agree on the extent to which the former falls under the purview of the latter. These issues have therefore been handled with an overall lack of specificity.

This review points out that despite rhetorical attention, there is little jurisprudence and systematic integration of human rights related to women living with HIV in the context of SRH. As this review of the jurisprudence shows, there are clear gaps and areas of concern that have not yet been sufficiently addressed.

A number of critical human rights issues have not been well addressed, for example the economic independence and financial security of women living with HIV and its influence on their ability to exercise their sexual and reproductive rights [8]. The Global Commission on HIV and the Law noted that when women lack the protection of laws that recognize equal rights to property, they are more likely to be rendered economically dependent on, and susceptible to, control by their spouses in all domains, including their sexual lives [8].

Furthermore, while issues such as criminalization of SRH services are often dealt with by international, regional and national human rights bodies [33], a systematic analysis is often missing of issues related to misinformation, intimidation tactics and barriers faced by women living with HIV in access to SRH information and services [22]. Within the human rights jurisprudence, there are also persistent gaps in relation to dealing with specific SRH issues, such as unwanted pregnancy, cervical cancer screening and management for women living with HIV and safe abortion services [9]. The review also points towards gaps in relation to normative standards pertaining to fertility issues of women living with HIV generally, specifically in relation to the desire to have children [34], use of SRH services and advice from providers [35]. Overall issues related to training and preparedness of healthcare providers to provide services to women living with HIV are often inadequately dealt with in human rights jurisprudence. Evidence points towards the critical importance of these interventions [36].

## Conclusions

The last 20 years have seen improvements in SRH and human rights in many countries. This advance has been supported by awareness raised by women's health advocates, increasingly by youth groups, and also by organizations of health professionals [37]. In the HIV/AIDS area, the involvement of organizations of people living with HIV/AIDS is crucial to improve prevention and care. The advocacy done by women living with HIV has helped both in the normative development of standards related to women living with HIV and in the improvement of treatment and care at the country level [38]. However, after victories during the 1990s, whereby women's rights groups made strides by combatting opposition from social and political conservatives, recent years have seen the backsliding of gains made [37].

The right to SRH is an essential part of the right to health and is rooted in numerous international human rights instruments. Despite the development in international standards and jurisprudence, the full enjoyment of the right to SRH remains a distant goal for millions of people throughout the world. This analysis of key human rights principles shows that issues related to the human rights of women living with HIV regarding SRH have not been comprehensively dealt with by the UN or other human rights mechanisms. This leaves critical gaps in normative developments in this area, which often result in *ad hoc* integration of these issues into health policies and programming.

At the national level, governments have not dealt with many human rights principles and outcomes as part of their legal and policy response to the human rights of women living with HIV. For example, discrimination, stigma and prejudice against women living with HIV occur primarily within families, communities and healthcare facilities; however, these issues are not appropriately dealt with at the national level. National legislation rarely deals with issues related to availability, privacy and confidentiality, acceptability, quality of services and meaningful participation by the community of women living with HIV. Resulting policies lack human rights guarantees for women living with HIV. There is therefore a clear need for strengthening global, regional and national standard setting for this underserved population. Within the findings of different human rights bodies at the global, regional and national levels, it was found that the language used for articulation of recommendations and standards is often pejorative and stereotypical and does not take into account the health and human rights of women living with HIV.

Further work is also needed to strengthen normative standards at the country level and enhance accountability for the violations of human rights of women living with HIV. Clear normative guidance is needed at the global, regional and national levels to address the SRH and human rights needs of women living with HIV. This work should build on the work of organizations and groups of women living with HIV. Furthermore, regular monitoring of implementation of the recommendations by the UN Human Rights Council through its UPR Working Group and Special Procedure mechanism can help enhance accountability for the human rights of women living with HIV.

A promising vision has been created by the growing youth movement for SRH and rights and the potential for opening up larger alliances around sexual and bodily rights with HIV/AIDS activists, sex workers, people living with HIV and AIDS and human rights organizations [38]. Together these alliances can lead to a meaningful change in the lives of this vulnerable group [39].

## Supplementary Material

Advancing the sexual and reproductive health and human rights of women living with HIV: a review of UN, regional and national human rights norms and standards

Advancing the sexual and reproductive health and human rights of women living with HIV: a review of UN, regional and national human rights norms and standards
