# Supplementary material for: Advancing the sexual and reproductive health and human rights of women living with HIV: a review of UN, regional and national human rights norms and standards
Source: J Int AIDS Soc. 2015 Dec 1;18(6Suppl 5):20280. doi: 10.7448/IAS.18.6.20280 (PMC4672403; doi:10.7448/IAS.18.6.20280)
Supplement: Advancing the sexual and reproductive health and human rights of women living with HIV: a review of UN, regional and national human rights norms and standards [file JIAS-18-20280-s001.pdf]

**ANNEX I: SEARCH TERMS AND DATABASES**

OHCHR Universal Human Rights Index (http://uhri.ohchr.org/en/Search/Annotations)

- Keywords: "reproductive and/or sexual health" (search type "all of these words"); filter by affected persons: "women"/"persons with HIV"
- Keywords: "women with HIV" (search type "all of these words"); filter by special procedures
- Keywords: "women with HIV" (search type "all of these words"); filter by Treaty body
- Keywords: "women with HIV" (search type "all of these words; filter by UPR)
- Keywords: "reproductive and/or sexual health" (search type "any of these words); filter by affected persons "persons with HIV"; filter by UPR/treaty body/special rapporteur
- Keywords: "cervical/cancer" (search type "any of these words"); filter by affected persons "persons living with HIV/AIDS"
- Keywords: "infertility" (search type "any of these words"); filter by affected persons "persons living with HIV/AIDS"
- Keywords: "postpartum/newborn/antepartum" (search type "any of these words"); filter by affected persons "persons living with HIV/AIDS"
- Keywords: "family planning" (search type "any of these words"); filter by affected persons "persons living with HIV/AIDS"

Bayefsky.com (http://www.bayefsky.com/bytheme.php/id/1165)

- Search by theme or subject matter: maternity and pregnancy (concluding observations, articles, general comments and recommendations)
- Search by theme or subject matter: equality and discrimination → gender discrimination
- Search by theme or subject matter: health → family planning
- Search by theme or subject matter: abortion
- Search by theme or subject matter: access to information (concluding observations, articles, jurisprudence, general comments and recommendations)
- Search by theme or subject matter: education→ health education (dissemination of human rights information concluding observations, articles, general comments and recommendations)
- Search by theme or subject matter: HIV – AIDS → public health and prevention (concluding observations, jurisprudence, articles, general comments and recommendations)
- Search by theme or subject matter: pregnancy
- Search by theme or subject matter: women
- Search by theme or subject matter: effective remedies (concluding observations, articles, jurisprudence, general comments and recommendations)
- Search by theme or subject matter: adequate or decent standard of living (concluding observations, articles, general comments and recommendations)
- Search results: "women with HIV" + "reproductive health"
- Search results: "women with HIV" + "sexual health"
- Search results: "women with HIV" + abortion

University of Minnesota Human Rights Library
(http://www1.umn.edu/humanrts/google/localsearch.html)

- Human Rights Committees (Treaty Bodies Combined): “women living with HIV”; “women with HIV”; “sexual health” + “women with HIV; “ reproductive health” + “women with HIV”
- Human Rights Committee Decisions and views: “women living with HIV”
- Human Rights Committee General Comments: “women living with HIV”
- Human Rights Committee Country conclusions and observations: “women living with HIV”
- Committee on Economic, Social and Cultural Rights: “women living with HIV”; “sexual health”; “reproductive health”
- Committee on the Elimination of Discrimination Against Women: “women living with HIV”; “women with HIV”; “HIV/AIDS”; “sexually transmitted diseases”
- Committee Against Torture: “women living with HIV”; “sexual health”; “reproductive health”
- UN Human Rights Council: “women living with HIV”; “women with HIV”; “HIV/AIDS”; “sexually transmitted diseases”
- *Note: did not search Committee on the Rights of the Child document as had already collected significant number of findings regarding vertical transmission*

Universal Periodic Review (http://www.upr-info.org/database/)

- States Under Review “all”; Recommending state “all”; Issue “HIV/AIDS”; “all cycles”; “recommendations and voluntary pledges”; keywords: “sexual health/reproductive health”
- States Under Review “all”; Recommending state “all”; Issue “HIV/AIDS”; “all cycles”; “recommendations and voluntary pledges”; keywords: “maternal/health”
- States Under Review “all”; Recommending state “all”; Issue “HIV/AIDS”; “all cycles”; “recommendations and voluntary pledges”; keywords: “sexual right” OR “reproductive right”
- States Under Review “all”; Recommending state “all”; Issue “HIV/AIDS”; “all cycles”; “recommendations and voluntary pledges”; keywords: “sexual health/reproductive health”
- States Under Review “all”; Recommending state “all”; Issue “HIV/AIDS”; “all cycles”; “recommendations and voluntary pledges”; keywords: “unsafe/involuntary abortion/steriliz(ation)”
- States Under Review “all”; Recommending state “all”; Issue “HIV/AIDS”; “all cycles”; “recommendations and voluntary pledges”; keywords: “cervical/cancer” / “breast cancer”
- States Under Review “all”; Recommending state “all”; Issue “HIV/AIDS”; “all cycles”; “recommendations and voluntary pledges”; keywords: “family planning” / “contracept(ion)” / “infertile(ity)” / “gynaecological” / “postpartum” / “newborn” / “antenatal”
