# Supplementary material for: Advancing the sexual and reproductive health and human rights of women living with HIV: a review of UN, regional and national human rights norms and standards
Source: J Int AIDS Soc. 2015 Dec 1;18(6Suppl 5):20280. doi: 10.7448/IAS.18.6.20280 (PMC4672403; doi:10.7448/IAS.18.6.20280)
Supplement: Advancing the sexual and reproductive health and human rights of women living with HIV: a review of UN, regional and national human rights norms and standards [file JIAS-18-20280-s002.pdf]

ANNEX II: SEARCH TERMS AND DATABASES

OAS/IACHR (www.oas.org/en/iachr)

- Keywords: “women” / “persons with HIV”; “living with HIV” (reports; decisions; resolutions)
- Keywords: “reproductive and/or sexual health” (reports; decisions; resolutions)
- Keywords: “infertility”; “persons living with HIV/AIDS” (reports; decisions; resolutions)
- Keywords: “postpartum/newborn/antepartum”; “persons living with HIV/AIDS” (reports; decisions; resolutions)
- Keywords: “family planning”; “persons living with HIV/AIDS (reports; decisions; resolutions)
- Keywords: “gender discrimination”; “HIV” (reports; decisions; resolutions)
- Keywords: “abortion”; “HIV” (reports; decisions; resolutions)
- Keywords: “access to information”; “HIV” (reports; decisions; resolutions)
- Keywords: “steriliz(ation)”; “HIV” (reports; decisions; resolutions)

AU / ACHPR (www.achpr.org/search/)

- Keywords: “women” / “persons with HIV”; “living with HIV” (documents search type: “Resolutions”; “Commission Activity Reports”; “State Reports”; “Concluding Observations”)
- Keywords: “reproductive and/or sexual health” (documents search type: “Resolutions”; “Commission Activity Reports”; “State Reports”; “Concluding Observations”)
- Keywords: “infertility”; “persons living with HIV/AIDS” (documents search type: “Resolutions”; “Commission Activity Reports”; “State Reports”; “Concluding Observations”)
- Keywords: “postpartum/newborn/antepartum”; “persons living with HIV/AIDS” (documents search type: “Resolutions”; “Commission Activity Reports”; “State Reports”; “Concluding Observations”)
- Keywords: “family planning”; “persons living with HIV/AIDS” (documents search type: “Resolutions”; “Commission Activity Reports”; “State Reports”; “Concluding Observations”)
- Keywords: “gender discrimination”; “HIV” (documents search type: “Resolutions”; “Commission Activity Reports”; “State Reports”; “Concluding Observations”)
- Keywords: “abortion”; “HIV” (documents search type: “Resolutions”; “Commission Activity Reports”; “State Reports”; “Concluding Observations”)
- Keywords: “access to information”; “HIV” (documents search type: “Resolutions”; “Commission Activity Reports”; “State Reports”; “Concluding Observations”)
- Keywords: “steriliz(ation)”; “HIV” (documents search type: “Resolutions”; “Commission Activity Reports”; “State Reports”; “Concluding Observations”)

CoE / Commissioner for Human Rights (www.coe.int)

- Keywords: “women” / “persons with HIV”; “living with HIV”
- Keywords: “reproductive and/or sexual health”
- Keywords: “infertility”; “persons living with HIV/AIDS”
- Keywords: “postpartum/newborn/antepartum”; “persons living with HIV/AIDS”
- Keywords: “family planning”; “HIV/AIDS”
- Keywords: “gender discrimination”; “HIV”
- Keywords: “abortion”; “HIV”
- Keywords: “access to information”; “HIV”

- Keywords: “steriliz(ation)”; “HIV”

National judgments:

- Keywords: “women” / “persons with HIV”; “living with HIV”
- Keywords: “reproductive and/or sexual health”
- Keywords: “family planning”; “HIV/AIDS”
- Keywords: “abortion”; “HIV”
- Keywords: “steriliz(ation)”; “HIV”
